# Communicating Treatment-Related Symptoms Using Passively Collected Data and Satisfaction/Loyalty Ratings: Exploratory Study

**DOI:** 10.2196/29292

**Published:** 2022-04-29

**Authors:** Ian Kudel, Toni Perry

**Affiliations:** 1 Varian, a Siemens Healthineers Company Palo Alto, CA United States

**Keywords:** electronic patient-reported outcomes, ePRO, cancer, symptoms, health-related quality of life

## Abstract

**Background:**

Electronic patient-reported outcomes’ real time communication of treatment-related symptoms is increasingly associated with better outcomes including longer survival and less health care resource use, but the primary method of collecting this information, static questionnaires, has not evolved.

**Objective:**

The aim of this paper is to describe the use of Noona’s three methods of communicating treatment-related symptoms, which are as follows: (1) Noona symptom questionnaires (NSQ), which incorporate branching logic; (2) a diary; and (3) secure messaging, the last two of which have NSQ reporting functionality. It also aims to explore, using multivariable analyses, whether patients find value using these features.

**Methods:**

Noona users (N=1081) who have an active account for more than 30 days, who responded to the satisfaction/loyalty item, and who were undergoing active cancer treatment (systemic or radiotherapy) in the United States were included in this study. All study data were collected via software embedded within Noona code. This includes metadata, patient activities (measured in clicks), and responses to a satisfaction/loyalty question (“How likely are you to recommend Noona to another patient”) displayed on the Noona home page.

**Results:**

Noona users expressed a high degree of satisfaction/loyalty when asked to rate how likely they would recommend Noona to another patient. Multivariable analyses indicate small but significant effects for some of the analyses. Use of NSQs were significantly related to satisfaction/loyalty, users of NSQs had significantly higher satisfaction/loyalty than those who did not use any, and secure communication use was significantly higher for those who rated the app highly compared to those who did not. These relationships will likely be further explicated with the use of satisfaction/loyalty questions that focus specifically on feature use.

**Conclusions:**

Noona is well liked by respondents, and exploratory multivariable analyses demonstrate the potential for using passively and minimally invasive data to demonstrate value.

## Introduction

For over 30 years, the systematic collection of patients’ experiences via electronic administration of static measures have been used to facilitate cancer treatment planning [1]. The current generation of devices are powerful, portable, internet accessible, and increasingly loaded with sophisticated capabilities and features. This has enabled real time patient-clinical care team communication of treatment-related symptoms. However, software interfaces have been described as “rudimentary” [2], and the primary method of collecting patient-reported data has been relatively static, which may impede patient engagement and long-term use.

Generally, research testing the electronic patient-reported outcomes (ePROs) impact can be divided into three groups. The first, randomized controlled trials, have consistently demonstrated the benefits of using this software. Basch et al [3] found those who used a web-based application and rated their treatment experiences using a 12-item questionnaire incorporating Common Terminology Criteria for Adverse Events (CTCAE) items remained on chemotherapy longer, reported significantly slower declines in health-related quality of life, used emergency department services less, and survived longer than those who used standard care. Two other studies have combined patient-reported treatment information with algorithms to improve functionality and better optimize clinical care. The first, a trial focusing on patients with lung cancer, found those in the treatment arm, which involved patients reporting symptoms via a weekly questionnaire, informed the computed tomography scan schedule. As a result, they lived longer and required fewer imaging tests compared to those receiving standard care (reporting symptoms to the family doctor or oncologist and attending regularly scheduled imaging appointments) [4]. The second study found that those who use an app that combines online symptom self-reporting with a clinical algorithm to generate automated advice to facilitate symptom self-management reported less decrement in physical well-being at 12 weeks and improved health-related quality of life in study participants at 18 weeks compared to standard care [5].

Real-world studies have demonstrated that an ePRO can facilitate reporting of common treatment symptoms (eg, tiredness, fatigue, and anxiety) compared with standard medical records [6], and a separate study found population level benefit in patients with cancer, including improved 1-, 3-, and 5-year survival [7]. A third group, feasibility studies, has focused on testing ePRO solutions in various patient populations in which little or no ePRO evidence has been generated including radiotherapy [8], immunotherapy [9,10], surgery [11,12], and palliative care [13].

There has been an increasing recognition that ePRO-associated benefits can only be accrued through durable patient engagement [14] and that current methods can be improved [15]. However, more interactive, engaging, and personalized designs can only be achieved by understanding user behavior patterns [14]. Varian Medical System’s ePRO platform, Noona, is a United States Food and Drug Administration Class 1 device. It is a multifunction software that includes three modalities that can be used to communicate and track treatment-related symptoms via CTCAE-based [3] Noona symptom questionnaires (NSQ) to the clinical care team in real time. They are (1) questionnaires administered at regular intervals, which are also available for ad hoc reporting; (2) a diary; and (3) secure messaging, the last two of which incorporate NSQ tracking and reporting functionality. Between November 2020 and January 2021, Noona implemented a code within its software that collects objective app use information and assesses satisfaction and loyalty using a single, minimally invasive question, “How likely are you to recommend Noona to another patient.” The patients responded using an 11-point visual analog scale [16]. Variations of this question and the associated statistic, Net Promoter Score [16], are used by two-thirds of Fortune 1000 companies to measure customer satisfaction and loyalty [17]; they have also been used within the field of medicine to gauge the quality of various medical services [18-20], implementation of a telehealth system [21], and evaluation of software developed for patients with cancer [22,23] and cancer survivors [24].

Previous research has demonstrated the efficacy of using electronic devices to collect passive exercise data used by patients with cancer generally [25-27] and that such information is associated with self-reported treatment symptoms [28]. This information can be easily collected without inconveniencing patients or clinical staff; however, it is not clear whether such data, along with the minimally invasive collection of satisfaction/loyalty ratings, can be used to demonstrate ePRO value. Thus, the goal of this real-world study is to report how Noona users employ the three Noona communication and tracking features (scheduled and ad hoc CTCAE-based NSQs; a diary with NSQ tracking functionality; and secure messaging). This study also aims to rate app satisfaction/loyalty and explore, using multivariable analyses, whether patients find value using these features. Our hypotheses are that, regardless of app features or construction, users should value the most important component of communication/tracking of treatment-related symptoms. Thus, the first set of analyses will explore the association between communication and tracking features and satisfaction/loyalty. Next, analyses will test whether those who use these features report greater satisfaction/loyalty than those who do not. The last set will determine whether there is a difference between those who rate the app highly and those who do not, regarding using the three communication and tracking features.

## Methods

### Noona, Participants, and Procedure

Noona is an ePRO that has been installed in over 100 oncology clinics across 10 countries. It is currently available in 8 languages and has over 100,000 active users. Clinical staff at each site onboard patients and assist them with creating a patient profile. The participants (n=1081) in this study were experienced Noona users, which is defined as users who have an active account for more than 30 days, who responded to the satisfaction/loyalty item, and who were undergoing active cancer treatment (systemic or radiotherapy) in the United States between January 2021 (the first-day objective data and patient satisfaction/loyalty were both collected) and March 17, 2021 (when the data were downloaded and analyzed).

All study data (metadata, patient activities measured in clicks, and satisfaction/loyalty scores) were collected via software embedded within Noona code. Study information was passively collected. The satisfaction/loyalty question is administered randomly every 3 months. It pops up on the Noona home page, and users can either respond to it or opt out.

### Ethical Considerations

Data were used for quality improvement purposes and thus not submitted for IRB approval; however, Noona clearly communicates patient rights when they sign on to use the app. Specifically, when creating an account, they have the option of authorizing data sharing and are informed of those rights. This includes a statement that Noona collects information for several purposes including data analysis for resource optimization, which is the case for this study. Additionally, Noona ensures that the data used for any analyses will be deidentified. Further, patients are told that if they choose not to share their data, it will not affect the care received from the health care provider, eligibility for benefits, or payment for health care, and that they will still have access to the app. Patients are informed that they can revoke this authorization at any time prior to expiration by contacting Noona (info@Noona.com). Finally, users are informed that this authorization ends upon deletion of the Noona account. When this occurs, any data collected by Noona will remain with Noona, but the health care provider will not further disclose any health information concerning the patient to Noona.

### Measures

#### Days Active

Noona reports the number of days since the patient activated an account. It is a continuous variable and is used as a covariate in this study.

#### Time on the App

Noona measures use in the number of total minutes the app was used since activation. It is a continuous variable and is used as a covariate in this study.

#### Age

Approximate patient age was calculated by subtracting the current year (2021) from the patient’s birth year, which was extracted as metadata.

### Device

Noona captures the operating system of the device that the patients last used to log into the system (eg, Windows or iOS). This information was used to create a dichotomous item representing the device type—computer, smartphone, or tablet. This variable was used a covariate in this study.

### Satisfaction/Loyalty

Noona assesses satisfaction/loyalty by asking users to answer the question, “How likely are you to recommend Noona to another patient?” using an 11-point visual analog scale (ranging from 0 to 10) with the anchors “Unlikely” and “Very Likely” at opposite ends of the scale. The respondents click on the rating and then submit it. The information is often grouped into three categories. Patients who rated the app from 0 to 6 were categorized as “Detractors,” those who rated it 7 or 8 where considered “Passive,” and those who rated the app 9 or 10 were characterized as “Promoters” [16]. For this study, patient responses were reported using this taxonomy or the original 11-point scale.

### Noona Symptom Questionnaires

NSQs were created by an advisory board of physicians who have clinical and research expertise within the specific treatment modality. NSQs are used to report treatment symptoms. The specific questionnaire is predicated on the treatment regimen. For example, patients receiving systemic therapy may receive the Chemotherapy-18 module, while those receiving radiotherapy for a pelvic cancer would be administered NSQs with that content. All NSQs include CTCAE-derived items in which patients can report 3 grades of severity (mild, moderate, and severe) and branching logic which reduces patient burden by eliminating the need to respond to items that are not relevant to the patient. Any responses that meet prespecified criteria will trigger alerts that can be viewed by the clinical care team. In turn, the team responds by suggesting an intervention, or in the case of an emergent concern, it instructs the patient to seek immediate medical attention. Some sites may assign a questionnaire by sending notifications asking patients to complete the questionnaire at prespecified times, though patients always have the option of using it any time. In this study, all clicks within this Noona feature are recorded and represent its use. Thus, a patient who clicked on this section once will have a score of 1, and another who clicked on this area 10 times will have a score of 10. Depending on the analysis, this variable was either an outcome or predictor variable.

### Diary

Noona’s diary feature gives patients the opportunity to save personal clinical and nonclinical information that can be used for a range of purposes including symptom tracking over time. However, this study focuses on the symptom-reporting component, which can be used to communicate with the clinical team in specific circumstances. Thus, similar to the NSQs, every click within this portion of Noona is recorded and represents a single use. Therefore, a patient who clicked on that section once will have a score of 1, and another who clicked within this area 10 times will have a score of 10. Depending on the analysis, this variable was either an outcome or predictor variable.

### Secure Messaging

This feature gives patients the ability to directly communicate with the clinical care team regarding clinically relevant and nonrelevant issues. Since this study focuses on clinically relevant issues, only those data are included. Similar to the other two features, every click is recorded and represents a single use. Thus, a patient who clicked on this section once will have a score of 1, and another who clicked on this area 10 times will have a score of 10. Depending on the analysis, this variable was either an outcome or predictor variable.

### Feature Preference

The patients were sorted into 1 of 4 categories (“None,” “NSQ,” “Diary,” and “Secure messaging”) based on the feature they used most often (defined by number of clicks). Note that those who did not use any of the 3 specific features were included in the “None” category.

### Analyses

Four sets of analyses are conducted for this study. The first set used descriptive statistics to report all study variables. Categorical variables were reported using count and percentage, and continuous variables are reported using means and standard deviations.

The remaining analyses are exploratory and used generalized linear models (GLMs), specifying a negative binomial distribution and a log link function, to test the relationship between the use of the three communication and tracking features and satisfaction/loyalty in accordance with a priori hypotheses. Additionally, the grand estimated marginal mean (the mean response for each factor, calculated as least-squares means presented at the mean of the covariates) and estimated marginal means were calculated using a maximum likelihood algorithm and are reported in their original metric.

The first hypothesis was tested by using separate GLMs to ascertain whether a symptom or tracking feature was associated with satisfaction/loyalty, controlling for Noona use (days active and time on app), age, and device. The next hypothesis, that patients who do not use any of the tracking features will report lower satisfaction/loyalty scores compared with those who have a feature preference, was assessed by testing the association between the categorical variable feature preference and satisfaction/loyalty scores, controlling for Noona use (days active and time on app), age, and device. The reference category for the feature preference variable was “None.” The final hypothesis was tested using separate GLMs to ascertain whether the hypothesis that Detractors use each of the three symptoms’ reporting and tracking features less than Promoters, controlling for Noona use (days active and time on app), age, and device. The covariates included in the analyses were not the primary focus of the study; thus, only those that were significant predictors across all models are reported at the end of the section to identify trends more easily.

## Results

The participants (Table 1 and Table 2) were generally older (mean age 65.16 years, SD 12.29), with active accounts for approximately three-quarters of a year (mean 285.22 days, SD 173.78), spent approximately 1 hour and 15 minutes using Noona (mean 76.41 minutes, SD 77.28), and were more likely to use smartphones or tablets (n=786; 72.4%) the last time they logged in. The overall satisfaction/loyalty rating was 8.05 (SD 2.91).

**Table 1:** Descriptive data of categorical variables.

Variables		Values, n (%)
**Device**		
	Computer	295 (27.16)
	Smartphone	786 (72.38)
**Satisfaction/loyalty groupings**		
	Detractors	227 (20.90)
	Passive	187 (17.22)
	Promoter	672 (61.88)

**Table 2:** Descriptive data of continuous variables.

Characteristics	Values
Age (years), mean (SD)	65.16 (12.29)
Duration since activation (days), mean (SD)	285.22 (173.78)
Time on app (min), mean (SD)	76.41 (77.28)
Satisfaction/loyalty, mean (SD)	8.05 (2.91)
NSQ^a^, mean (SD)	1.26 (2.64)
Diary, mean (SD)	0.78 (2.21)
Secure messaging	0.69 (1.80)

^a^NSQ: Noona symptom questionnaires.

**Figure 1:**
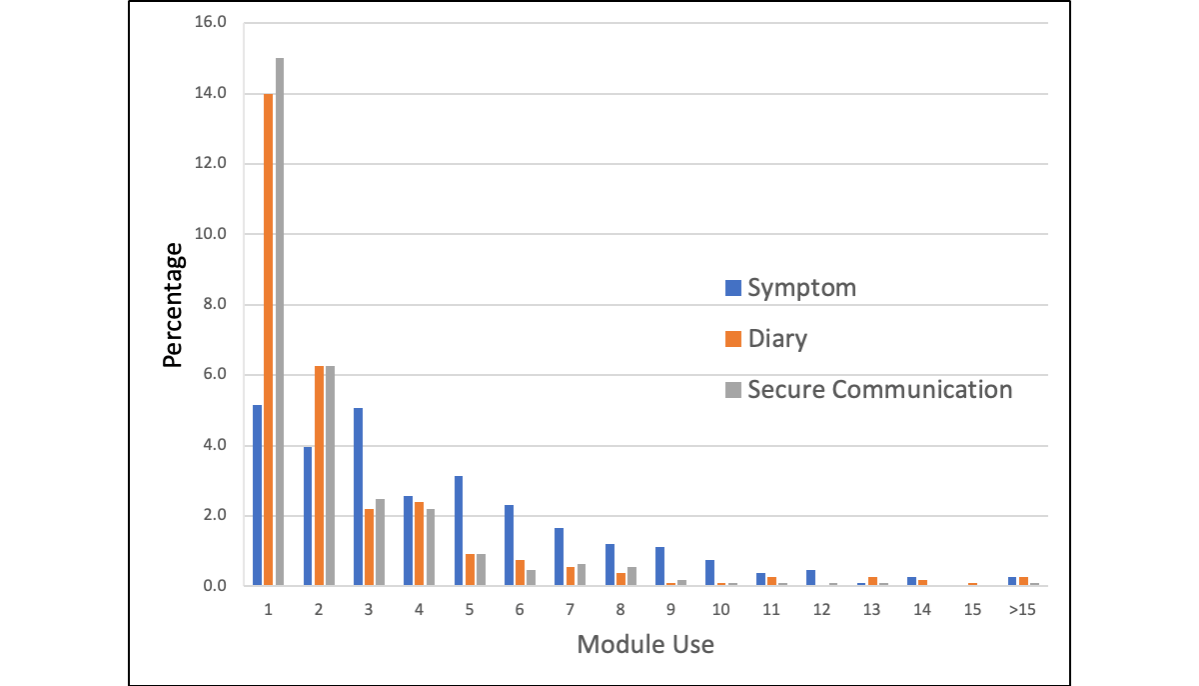
Patients' use of symptom, diary, and secure communication modalities by clicks.

Of the total 1081 patients, 308 (28.36%) patients used the NQS, 312 (28.73%) used the diary, and 317 (29.19%) used secure communication modalities, respectively. Overall use ranged between 1 and 33 times (Figure 1). Patients tended to use NQS portions of the application most (mean 1.26 clicks, SD 2.64), followed by the diary (mean 0.78 clicks, SD 2.21), and secure messaging (mean 0.69 clicks, SD 1.80). Over half of the participants gave a satisfaction/loyalty rating to Noona. Promoters (scores of 9 or 10: n=672, 61.88%; Table 3) comprised more than 60% of the sample compared to Passives (scores of 7 or 8: n=187, 17.22%) and Detractors (scores between 0 and 6: n=277, 20.90%). The mean rating was 8.05 (SD 2.91).

The GLMs testing the relationship between NSQ use and satisfaction/loyalty were significant (*B*=0.01, *P*=.05; Table 4). This indicates that, for every NSQ module click, a 0.01 increase in satisfaction/loyalty score is predicted. The grand estimated marginal mean was 7.91. The confidence intervals were within a tenth of a point indicating a high degree of accuracy. The other two models did not find a significant relationship between diary and secure messaging use and patient satisfaction.

The next analysis found that patients who used the NSQ most often reported significantly higher satisfaction/loyalty scores compared to those who did not use any of the three features (*B*=0.71, *P*=.02; Table 5).

**Table 3:** Participants’ satisfaction/loyalty scores.

Participants and NPS^a^		Frequency	Percentage
**Promoters**			
	0	57	5.25
	1	19	1.75
	2	21	1.93
	3	15	1.38
	4	19	1.75
	5	75	6.91
	6	21	1.93
**Passive**			
	7	53	4.88
	8	134	12.34
**Detractors**			
	9	99	9.12
	10	573	52.76

^a^NPS: Net Promoter Score.

**Table 4:** Generalized linear models testing the relationship between accessing new modules and satisfaction/loyalty.

Modalities and variables		Values					
		*B*	SE	*P* value	Exp (*B*)	95% CI for odds ratios	
						Lower	Upper
**NSQ^a^**							
	App time	0	0	.47	1.00	1.00	1.00
	Days since activation	0	0	.29	1.00	1.00	1.00
	Age	0	0	<.001^b^	1.00	1.00	1.00
	Device	–0.07	0.03	.01^b^	0.93	0.89	0.98
	NSQ use	0.01	0	.05^b^	1.01	1.00	1.02
**Diary**							
	App time	0	0	.20	1.00	1.00	1.00
	Days active	0	0	.28	1.00	1.00	1.00
	Age	0	0	<.001^b^	1.00	1.00	1.00
	Device	–0.07	0.03	.01^b^	0.94	0.89	0.98
	Diary	0	0.01	.66	1.00	0.99	1.01
**Secure communication**							
	App time	0	0	.26	1.00	1.00	1.00
	Days since activation	0	0	.28	1.00	1.00	1.00
	Age	0	0	.01^b^	1.00	1.00	1.00
	Device	–0.07	0.03	.01^b^	0.94	0.89	0.98
	Secure messaging	0	0.01	.47	1.00	0.99	1.02

^a^NSQ: Noona symptom questionnaires.

^b^*P*<.05

**Table 5:** Generalized linear models testing the relationship between feature preference and satisfaction/loyalty (“None” was the reference group).

Variables	Values					
	*B*	SE	*P* value	Exp (*B*)	95% CI for odds ratios	
					Lower	Upper
App time	0	0	.40	1.00	1.00	1.00
Days since activation	0	0	.38	1.00	1.00	1.00
Age	0	0	.99^a^	1.00	1.00	1.00
Device	–0.06	0.03	.02^a^	0.94	0.89	0.99
Secure communication	0.03	0.03	.39	1.03	0.97	1.10
Diary	0.03	0.03	.40	1.03	0.96	1.10
NSQ^b^	0.07	0.03	.02^a^	1.07	1.01	1.14

^a^*P*<.05

^b^NSQ: Noona symptom questionnaires.

The grand estimated marginal mean was 7.94. The estimated marginal means for NSQ (8.26) was 0.57 points higher than the “None” category (7.69). The two other features (diary=7.91; secure messaging=7.91) were also higher than “None.” The confidence intervals were within a tenth of a point, indicating a high degree of accuracy. The final set of analyses (Table 6) found that Detractors and Promoters significantly differ in their use of the secure communication feature (*B*=1.307, *P*=.04). The grand marginal mean was 0.11 clicks, and the estimated marginal mean was 0.13 clicks for Promoters and 0.11 for Detractors. The confidence intervals were within a tenth of a point, indicating a high degree of accuracy.

Examination of the covariates found a general trend for age; it was a significant predator in all models, but the relationship was small. For example, in the model testing the relationship between NSQ and satisfaction/loyalty, for every minute of app use there was less than a 0.01 increase in clicks predicted. Additionally, the device patients used was also a significant predictor across all models, but the relationship differed depending on the model. For example, for all three models testing the relationship between communication and tracking features and satisfaction/loyalty, patients found consistent estimated marginal means were higher for smartphone or tablet use (8.14) compared with computers (7.81). In the analyses, testing whether Detractors and Promoters differentially predicted the use of the treatment symptom and tracking features, we found that for the models predicting NSQ and secure messaging, the estimated marginal means were higher for smartphone or tablet use (0.25 and 0.13, respectively) compared with computer (0.10 and 0.20, respectively). It was reversed for the model that included the diary (computer=0.11; tablet or smartphone=0.12).

**Table 6:** Generalized linear models comparing those with low and high satisfaction on communication and tracking features (Detractors was the reference group).

Variables			Values										
			*B*		SE		*P* value		Exp (*B*)		95% CI for odds ratios		
											Lower		Upper
**NSQ^a^**													
	App time	0.01		0		<.001^b^		1.01		1.01		1.01	
	Days since activation	0		0		.59		1.00		1.00		1.00	
	Age	0.02		0		<.001^b^		1.02		1.01		1.03	
	Device	0.22		0.11		.04^b^		1.25		1.01		1.53	
	Promoters	0.12		0.11		.28		1.13		0.91		1.41	
**Diary**													
	App time	0.01		0		<.001^b^		1.01		1.01		1.01	
	Days since activation	0		0		.01^b^		1.00		1.00		1.00	
	Age	–0.03		0		<.001^b^		0.97		0.96		0.98	
	Device	–0.17		0.13		.21		0.85		0.65		1.10	
	Promoters	0.11		0.14		.43		1.11		0.85		1.45	
**Secure communication**													
	App time	0.01		0		<.001^b^		1.01		1.01		1.01	
	Days since activation	0		0		.23		1.00		1.00		1.00	
	Age	–0.03		0		<.001^b^		0.97		0.96		0.98	
	Device	0.22		0.13		.10^b^		1.24		0.96		1.60	
	Promoters	0.23		0.14		.10^b^		1.26		0.96		1.66	

^a^NSQ: Noona symptom questionnaires.

^b^*P*<.05

## Discussion

Real time reporting of treatment symptoms via ePROs will increasingly become a critical component of cancer treatment because patients better recognize symptoms compared with providers [29,30]. There is increasing evidence that ePRO use positively impacts critical outcomes (eg, mortality) [4,5,31], and it will eventually be required for some reimbursement [15]. Therefore, real-world evidence demonstrating patients’ use and satisfaction with ePRO software will be a necessary requirement for all stakeholders (patients, providers, and payers) who want to simultaneously mitigate patient distress and realize cost savings. Noona includes, among an array of features, three methods of communicating and tracking treatment-related symptoms that distinguish it among other ePROs and electronic platforms. The addition of capabilities to collect objective app use and satisfaction/loyalty with minimal patient burden is the veritable “win-win” scenario. Certainly, this information can be used descriptively, but its ability to produce real-world evidence, such as a demonstration that the use of these tracking features is associated with patient satisfaction/loyalty, can yield deeper understanding of how patients use and value the app.

An incontrovertible finding is that patients like the app; more than half (n=570, 52.76%) gave it the maximum score of 10, and 61.98% (n=670) rated it a 9 or 10. The exploratory multivariate analyses demonstrate some small but significant relationships between objective data use of the three communication modalities in the form of clicks and responses to an item assessing Noona satisfaction/loyalty. They include the findings that NSQ use was a significant predictor of satisfaction/loyalty scores; patients using the NSQ reported significantly higher satisfaction/loyalty scores than those who did not use one of the three Noona communication features; and Promoters used the secure-messaging modality more than Detractors. In general, we think these exploratory analyses are successful because, by making some slight adjustments, it is relatively easy to refine the satisfaction/loyalty item so that respondents can focus on these features to guide ratings rather than other potential facets of the app. This will also likely resolve the obvious ceiling effect—patients rated the application so highly (over 50% reported a score of 10) that it reduced data variability, which also negatively impacting the analyses.

While we see great potential for the use of Net Promoter Scores, the data presented in our study have limitations. For example, we are not able to include more personal or clinically relevant data because they are not embedded within Noona. Additionally, we made some assumptions regarding the relationship between clicks and feature use, which future research may find to be suboptimal.

## Abbreviations

CTCAE: Common Terminology Criteria for Adverse Events
ePRO: electronic patient-reported outcomes
GLM: generalized linear model
NSQ: Noona symptom questionnaires
